# Advances in Research on *Panax Ginseng*-Derived EV-like Particles

**DOI:** 10.3390/ijms27188320

**Published:** 2026-09-18

**Authors:** Qi Yan, Tianxia Sun, Enpeng Wang

**Affiliations:** Jilin Ginseng Academy, Changchun University of Chinese Medicine, Changchun 130117, China

**Keywords:** *Panax ginseng*-derived EV-like particles, isolation, drug delivery, ginseng, vesicles

## Abstract

*Panax ginseng*-derived EV-like particles (PG-EVLPs) are nanoscale lipid bilayer vesicles extracted from ginseng roots, stems, leaves, or cell cultures, with diameters typically between 50 and 200 nm. Available preclinical studies have demonstrated that PG-EVLPs exhibit favorable in vitro and in vivo biocompatibility and short-term safety, without obvious hemolysis, cytotoxicity or organ damage, making them an active area of research at the intersection of traditional Chinese medicine and nanomedicine. This review covers separation and purification methods, physicochemical characterization techniques, bioactive component composition, and pharmacological mechanisms of PG-EVLPs. It also evaluates several delivery strategies, including natural nanocarrier delivery, engineering modification, and hydrogel composite systems, and discusses current challenges in standardization, safety assessment, and clinical translation.

## 1. Introduction

*Panax ginseng C. A. Meyer*, a cornerstone of traditional Chinese medicine, has a medicinal history spanning millennia and is widely used across Asian countries for enhancing immunity, combating fatigue, retarding aging, and treating diverse diseases [1]. Modern pharmacological research has established that the therapeutic basis of ginseng resides principally in its rich chemical repertoire, which includes ginsenosides, polysaccharides, volatile oils, peptides, and amino acids [2]. Among these, ginsenosides represent the most prominent active constituents; over 270 saponins have been identified to date, exhibiting anti-inflammatory, antitumor, immunomodulatory, and cardioprotective activities [3,4,5]. Ginsengs of different geographical origins and cultivars, notably Asian ginseng versus American ginseng, differ markedly in chemical profile and pharmacological properties [6,7], while the transformation of saponin constituents during red ginseng processing further diversifies its pharmacological substrate [8]. Conventional ginseng extracts, however, suffer from limited bioavailability, poor targeting specificity, and inadequate stability, prompting the search for more efficient delivery systems for active compounds.

Extracellular vesicles (EVs) is the umbrella term for naturally released membrane particles. The term “exosome” conventionally implies endosomal (MVB) biogenesis, which has not been experimentally demonstrated for most isolated plants. Ginseng-derived vesicles have been described under various terminology in current studies, including ginseng-derived extracellular vesicles (GEVs), ginseng-derived nanoparticles (GDNPs), ginseng exosome-like nanovesicles (GENVs), and ginseng-derived nanovesicles (GDNs), reflecting the lack of a standardized nomenclature in this field. According to the MISEV2023 guidelines, biogenesis-related terms such as “exosome” should be discouraged unless the subcellular origin of the particles has been experimentally demonstrated for the specific source and condition [9]. To date, no experimental evidence has established the endosomal origin of ginseng-derived vesicles, and most preparations are obtained from juiced, homogenized, or cell-wall-broken root tissue, which yields a heterogeneous population of lipid bilayer-enclosed nanoparticles rather than a defined EV subtype. We therefore adopt the more cautious and operationally descriptive term “*Panax ginseng*-derived EV-like particles (PG-EVLPs)” throughout this review, following the nomenclature framework proposed by the expert consensus on Chinese herbal medicine-derived EV-like particles (CHM-EVLP), in which such particles are designated by the Latin name of the medicinal plant combined with “EVLP” [10]. Unless otherwise specified, PG-EVLPs collectively refers to lipid bilayer-enclosed nanoparticles isolated from ginseng.

Plant-derived exosome-like nanovesicle membrane structures, ubiquitous throughout the plant kingdom, encapsulate and protect proteins, nucleic acids, and secondary metabolites, exerting pharmacological effects through trans-species cross-kingdom communication. Compared with animal-derived exosomes, ELNs offer distinct advantages such as higher yield, lower production cost, reduced immunogenicity, and the ability to withstand gastrointestinal absorption, all of which contribute to their therapeutic potential [11,12,13]. Studies indicate that *Panax ginseng*-derived EV-like particles not only inherit the diverse pharmacological properties of ginseng, including anti-inflammatory, antioxidant, anti-aging, and anti-osteoporotic activities, but also exhibit unique advantages in drug delivery and disease treatment owing to their nanoscale dimensions and natural origin [14]. PG-EVLPs extracted from ginseng roots, for instance, effectively suppresses osteoclast differentiation and displays anti-osteoporotic effects in vivo [15]; oral administration ameliorates and protects against liver fibrosis and gut dysbiosis [16]; and ameliorates colitis progression by suppressing inflammatory cytokines [17]. This offers a novel perspective: plant-derived nanovesicles are retained during traditional Chinese medicine decoction, implying that they may constitute a previously neglected group of functional components in herbal water decoctions.

**Figure 1:**
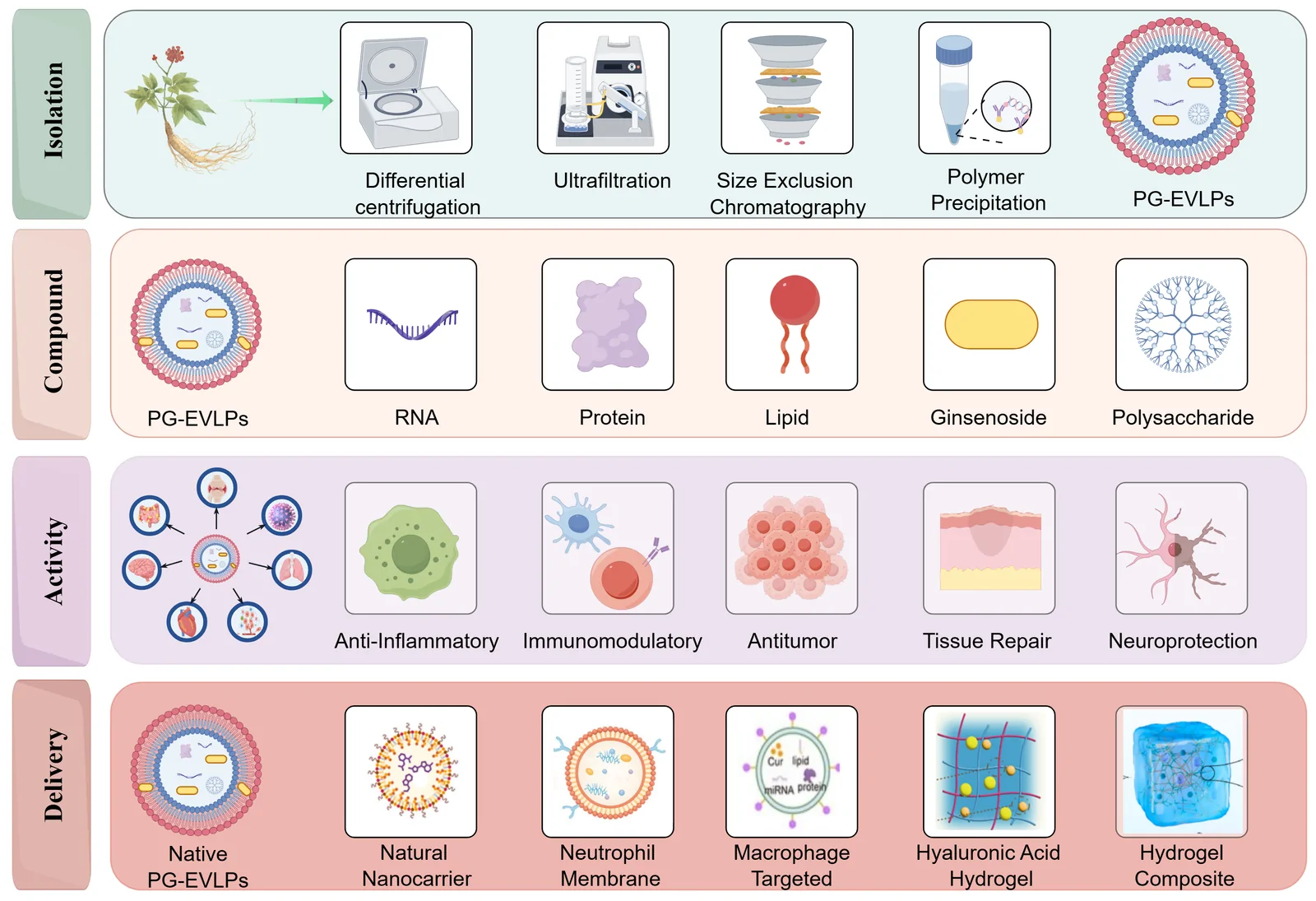
Isolation, compound composition, pharmacological activities and delivery applications of *Panax ginseng*-derived EV-like particles.

Several review articles have summarized plant-derived nanovesicles, but few integrate the full research chain of *Panax ginseng*-derived EV-like particles—from isolation and purification to bioactive components, pharmacological mechanisms, and delivery strategies. Most existing studies cover only one aspect and do not trace the connections across the field. We performed literature searches using Google Scholar with the search string: “(*Panax ginseng* OR ginseng) AND (exosome OR extracellular vesicle OR nanoparticle OR nanovesicle)”. This strategy was designed to comprehensively retrieve publications concerning the extraction and purification of PG-EVLPs, their bioactive components, biological applications, as well as relevant studies on PG-EVLPs. No explicit exclusion criteria were predefined. This narrative review addresses that gap by examining recent progress in PG-EVLPs across these four areas, analyzing current limitations, and proposing future research directions (Figure 1).

## 2. Isolation, Purification, and Physicochemical Characterization

### 2.1. Isolation and Purification Methods

Depending on the material and the extraction procedure, ginseng-derived vesicle preparations differ in composition and physicochemical properties. Following the MISEV2023 classification of extracellular particles (EPs), which distinguishes EVs, artificial cell-derived vesicles (ACDVs), and synthetic vesicles (SVs) [9], and referring to the practical recommendations for plant EV isolation [18], we classified ginseng-derived vesicles according to their production mode. Naturally secreted EVs, obtained from apoplastic wash fluid (AWF) of the culture medium of ginseng tissue cultures, were classified as EVs; vesicles obtained by methods that induce cell disruption, such as extrusion, juicing, and cell-wall breaking, were classified as ACDVs. ACDV-type preparations comprise a heterogeneous mixture that may contain naturally secreted EVs, intracellular vesicles, and cellular or organellar debris. Notably, naturally secreted ginseng EVs exhibited a smaller particle size, a lower zeta potential, and a lower concentration than artificial cell-derived vesicle preparations. A compilation of the extraction methods and the corresponding EP classification is provided in Table 1.

Differential centrifugation is the most fundamental and widely used method for extracting exosome-like nanovesicles from ginseng sources. Its underlying principle relies on the differential sedimentation coefficients of various components; by progressively increasing centrifugal force, impurities such as cell debris, large vesicles, and organelles are successively removed, ultimately yielding PG-EVLPs through precipitation under ultracentrifugation conditions. The typical procedure comprises the following steps: first, unbroken cells and tissue fragments are removed by low-speed centrifugation at 300× *g*; then larger organelles and microvesicles are eliminated by centrifugation at 2000× *g*; next, smaller organelle fragments are removed via centrifugation at 10,000× *g*; finally, PG-EVLPs are precipitated by ultracentrifugation at 100,000× *g* to 150,000× *g* [15]. Using this approach, researchers have successfully isolated exosomes from ginseng roots; transmission electron microscopy revealed a typical saucer-shaped bilayer membrane structure with a mean particle diameter of 129.7 nm [19]. While operationally straightforward and cost-effective, the method often yields products of insufficient purity, contaminated with protein aggregates and lipoprotein particles of comparable size. To address this limitation, differential centrifugation is frequently coupled with sucrose density gradient centrifugation, which achieves effective separation of high-purity vesicles through density stratification [20]. Although density gradient centrifugation markedly improves purity, the procedure is labor-intensive and time-consuming, and repeated centrifugation steps incur yield losses that constrain its applicability in large-scale preparation.

Ultrafiltration (UF) is a physical method based on membrane separation technology that directly retains *Panax ginseng*-derived EV-like particles (PG-EVLPs) using membranes with specific pore sizes (e.g., 100 nm or 200 nm), while allowing small molecular impurities, soluble proteins, and salts to pass through, thereby achieving preliminary enrichment of PG-EVLPs. This method operates under mild conditions without the need for organic solvents or high temperatures, maximally preserving the structural integrity and biological activity of the vesicles, and features rapid processing speed and ease of scale-up, making it suitable for large-scale production of PG-EVLPs [21].

Size exclusion chromatography (SEC) counts as a purification option for PG-EVLPs. It separates particles based on the molecular sieving effects of column resin. Researchers apply sequential filtration followed by SEC to isolate extracellular vesicles from ginseng callus culture supernatant. Nanovesicle tracking analysis detects nanovesicles ranging from 15 nm to 200 nm after SEC separation. This workflow can obtain functional PG-EVLP that promotes fibroblast migration. SEC can remove free soluble protein impurities. It helps preserve the native structure of nanovesicles [13,22]. Still, SEC has limited sample loading volume. It is suitable for further polishing after crude enrichment instead of initial enrichment.

The polymer precipitation method, particularly that utilizing polyethylene glycol (PEG), is a widely employed technique for isolating PG-EVLPs. Its fundamental principle involves altering the hydration state of the solution to reduce the solubility of PG-EVLPs, thereby promoting their aggregation and precipitation. The primary advantage of this approach lies in its simplicity, as it does not require expensive or complex equipment such as ultracentrifuges [23]. However, this method also has several significant limitations. First, as a polymer, PEG tends to remain in PG-EVLPs samples during precipitation. These residues can not only interfere with subsequent characterization of PG-EVLPs’ physicochemical properties, such as particle size, zeta potential, and morphological analysis, but may also affect their bioactivity assessment, given that PEG itself can exert non-specific effects on cells or organisms. Second, the polymer precipitation method exhibits poor selectivity; while precipitating PG-EVLPs, it often co-precipitates other non-vesicular impurities in the solution, including protein aggregates, polysaccharide complexes, and cell debris. Consequently, products obtained via simple PEG precipitation are typically crude extracts with significantly lower purity compared to those derived by ultracentrifugation, necessitating additional purification steps. It is therefore typically restricted to an initial enrichment step, necessitating combination with SEC or density gradient centrifugation.

This section compares four main PG-EVLPs separation technologies; their advantages and limitations are summarized in Table 1. Differential centrifugation and PEG precipitation are low-cost enrichment methods but yield low purity, while SEC-based methods produce high-purity vesicles at the cost of lower recovery. The choice of method should depend on specific research needs, and standardized separation protocols are needed to reduce variation in PG-EVLPs.

**Table 1:** Comparative analysis of different extraction and isolation methods.

Separation Methods	Differential Ultracentrifugation (dUC)	Tangential Flow Filtration (TFF)	Polymer Precipitation (PEG)	Membrane Filtration (MF)	Ultrafiltration (UF)	Gradient	Size-Exclusion Chromatography (SEC)
Separation Principle	Particles are separated by sedimentation rate through stepwise increases in centrifugal force. Low to medium centrifugation removes tissue residues and cell debris, while high-speed ultracentrifugation pellets nanovesicles.	Tangential-flow membrane filtration with a molecular weight cut-off (MWCO) involves sample flow parallel to the membrane. Small molecules and impurities pass through to the permeate, while nanovesicles larger than the MWCO are retained and concentrated (e.g., using a 100 kDa Minimate TFF Capsule).	Hydrophilic polymer (PEG 6000/ExoQuick) induces vesicle precipitation via steric exclusion, reducing the hydration layer and solubility, which promotes aggregation that can be collected by low-speed centrifugation.	Direct retention and permeation of particles by pore-size membranes (0.22/0.45 µm): vesicles are retained by size exclusion while smaller solutes pass through, resulting in a simple and high-yield operation.	Size exclusion using a MWCO semi-permeable membrane under centrifugal force allows small molecules (free drugs, proteins, dyes) to pass into the filtrate, while nanovesicles (greater than the MWCO) are retained and concentrated on the membrane.	Separation by buoyant density: vesicles reach isopycnic equilibrium in a sucrose/density gradient, with EVs enriched at the 30/45% interface.	Separation by hydrodynamic size occurs as large vesicles are excluded from the porous resin and elute first, while small proteins and metabolites enter the pores and elute later. This process effectively removes soluble protein contaminants while preserving bilayer integrity and bioactivity.
Procedure	(1) 2000× *g* for 20 min → 10,000× *g* for 60 min → 100,000× *g* for 90 min → sucrose gradient (8%/30%/45%/60%), 200,000× *g* for 90 min → 8%/30% interface [24,25,26]. (2) 400 → 800 → 15,000× *g* for 20 min → 100,000× *g* for 60 min → sucrose gradient (1 M/2 M), 150,000× *g* for 180 min [27,28] (3) 3000× *g* for 30 min → 10,000× *g* for 60 min → 150,000× *g* for 120 min → 0.22 µm filtration [29,30].	(1) 50 nm hollow-fiber TFF, retentate collection [16]. (2) 100 kDa MWCO + Masterflex pump, feed rate: 40.0 mL/min; pre-filtration through 1.22 µm, 0.44 µm, and 0.22 µm filters in series; diafiltration/concentration [14].	(1) PEG 6000: Filtrate + equal-volume PEG 6000, 4 °C for 24 h, pellet at 10,000× *g* for 1 h [31]. (2) ExoQuick: Crude extract (1000× *g* for 10 min → 3000× *g* for 20 min → 10,000× *g* for 60 min): ExoQuick = 5:1, 4 °C overnight, 1500× *g* at 4 °C for 30 min [32]. (3) ExoQuick-TC™ precipitation from ginseng cell culture supernatant [33].	220-nm and 30-nm track-etch membranes in series (self-made device), 200 µL/min [31]—“0.22 µm [29,30,34]“—“0.45 µm [35,36].	(1) 100 kDa ultrafiltration tube, 10,000× *g* for 15 min [37]. (2) 100 kDa ultrafiltration tube, 5000 rpm for 3 rounds [36].	(1) 8/30/45/60% (or 15/30/45/60%) sucrose gradient, 100,000–200,000× *g* for 60–120 min, followed by 30/45% interface collection [32,37,38,39,40,41,42,43,44]. (2) 27%/68% sucrose cushion followed by gradient [24,25,26]. (3) 1 M/2 M sucrose, 150,000× *g* for 2–3 h [27,28,45]. (4) 0.8 M/2 M sucrose cushion, 100,000× *g* for 2 h [35].	SEC on Sephacryl™ S-500 gel-filtration column (poly-prep columns); PBS elution at 4 °C; early fractions (vesicles) were collected, while later fractions (free proteins) were discarded [46].
Material Extraction	Ginseng callus/cell suspension culture supernatant [33,34,35]. Fresh ginseng root; buffer homogenization [25,27,28,32,41,43,44,45,47,48,49,50,51,52,53,54,55]. Fresh ginseng root; blender/grinding [16,24,30,38,39,56,57]. Fresh ginseng root; juice squeezing [37,40,42]. Fresh ginseng root; squeezing [20,26,58]. Fresh ginseng root; grinding [29,59].	Ginseng root: juice squeezing [14]. Ginseng root: blender/grinding [16].	Ginseng root cell suspension culture supernatant [33]. Ginseng root; juice squeezing [31]. Fresh ginseng root; buffer homogenization [32].	Ginseng root; juice squeezing [31]. Ginseng root; juice squeezing (hollow-fiber TFF) [14]. Fresh ginseng root; buffer homogenization [29,30,34]. Ginseng stem and leaf; buffer homogenization plus enzymatic digestion [36]. Ginseng cell-culture supernatant; 0.45 µm filtration (GcEVs) [35].	Ginseng root; juice squeezing [37]. Ginseng stem and leaf; buffer homogenization and enzymatic digestion [36].	Fresh ginseng root; buffer homogenization [28,32,41,47,48,53]. Fresh ginseng root; blender/grinding [15,19,24,25,27,38,39,45,57,60]. Fresh ginseng root; juice squeezing [26,31,35,37,40,42,43,51,59]. Ginseng stem and leaf; buffer homogenization + enzymatic digestion [36,44].	Fresh ginseng root; blender homogenization [46].
Extracellular Particle Classification	EV type [33,34,35]. ACDV-type [16,20,24,25,26,27,28,29,30,32,37,38,39,40,41,42,43,44,45,47,48,49,50,51,52,53,54,55,56,57,58,59].	ACDV type [14,16].	(1) EV type [33]. (2) ACDV-type [31,32].	ACDV type [29,30,31,34,35,36].	ACDV type [36,37].	ACDV type [15,19,24,25,26,27,28,31,32,35,36,37,38,39,40,41,42,43,44,45,47,48,51,53,57,59,60].	ACDV type [46].
Particle Size (nm)	EV type: 72–150 nm [33,34,35]. ACDV type: 29–499 nm (mostly 100–250 nm). Buffer homogenization: 100–256 nm [25,27,28,32,41,43,47,48,49,50,51,52,53,54,55]. Blender/grinding: 118–266 nm [16,30,38,39,56,57]. Juice squeezing: 69–345 nm [37,40,42]. Squeezing: 165–215 nm [20,26,58]. Grinding: 80–145 nm [29,59]. Horizontal comparison: GrEVs (root): 92.04 ± 4.85 nm vs. GcEVs (culture): 72 ± 25.95 nm [35]. dUC 168.7 ± 7.4 nm vs. TFF 228.7 ± 4.5 nm [16].	Juice squeezing: 159.5 nm [14]. Horizontal comparison: TFF: 228.7 ± 4.5 nm vs. dUC: 168.7 ± 7.4 nm [16].	EV type: ~150 nm [33]. ACDV type: Juice squeezing: 196.7 ± 10.78 nm [31]. Buffer homogenization: 42–389 nm (UC + ExoQuick combination) [32].	Horizontal comparison: MF among four methods (144.2 ± 6.46 nm) [31]; GcEVs: 72 ± 25.95 nm [35].	Juice squeezing: 76.87 nm [37]. Stem & leaf + enzyme: 127.3 nm [36].	ACDV type: 69–344.8 nm (mostly 100–250 nm) Horizontal comparison: Sucrose cushion among four methods (207.5 ± 7.69 nm) [31]. GrEVs: 92.04 ± 4.85 nm (0.8 M/2 M sucrose cushion) [35].	−108.5 to 132.6 nm [46].
Zeta Potential (mV)	Buffer homogenization: −12.3 to −39.0 mV [25,27,28,32,41,43,47,48,49,51,52,53,54]. Blender/grinding: −13.8 to −25.3 mV [24,30,38,39,57]. Juice squeezing: −19.0 to −25.4 mV [40,42]. Squeezing: −34.7 mV [20]. Grinding: −12.0 to −21.3 mV [29,59].	Not reported	Juice squeezing: −10.88 ± 0.27 mV [31]. Buffer homogenization: −28.88 mV [32].	−11.61 ± 0.60 mV [31].	−23.4 mV [36].	−8.14 mV to −39.0 mV (mostly −15 to −30 mV)	−20 to −25 mV [46]
Particle Concentration (particles/mL)	EV type: 6.94 × 10^10^ (GcEVs) [35] ACDV type: Buffer homogenization: 1.65 × 10^12^ to 3.12 × 10^12^ [32,53] Blender/grinding: 1.09 × 10^12^ ± 1.19 × 10^11^ to 2.05 × 10^13^ [24,26,57]. Horizontal comparison: dUC: 1.4 × 10^9^ vs. TFF: (3.8 × 10^6^) [16].	Juice squeezing: 3.9 × 10^12^ [14] Horizontal comparison: dUC: 1.4 × 10^9^ vs. TFF: (3.8 × 10^6^) [16].	Juice squeezing: 2.2 × 10^10^ [31]. Buffer homogenization: 1.08 × 10^12^ (UC + ExoQuick combination) [32].	1.5 × 10^11^ [31]. 6.94 × 10^10^ (GcEVs) [35].	1 × 10^12^/mL [36].	1 ×10^8^ to 10^13^ particles/mL Sucrose cushion + gradient: 2.05 × 10^13^ to 2.24 × 10^13^ [24,25]; dUC + gradient: 1.09 × 10^12^ [26].	3.72 × 10^11^ [46].
Yield	Grinding: 50 mg protein/kg fresh root [59]; 200–300 mg protein/kg root [28]. Blender/Grinding: 3.41 × 10^8^ particles/g [56]; 4.83 × 10^10^ particles/50 g root [30]. Horizontal Comparison: 0.4 mg protein/g ginseng (dUC). [16].	TFF concentrated retentate: 58.6 mL (0.7% volume, 150.5-fold) [14]. Total GNV yield: 0.4 mg protein/g ginseng (see dUC column; TFF has fewer particles) [16].	1.25 ± 0.03 µg/µL (protein concentration) [31].	2.78 ± 0.07 µg/µL (protein concentration) [31].	Not reported.	1 M/2 M:200–300 mg/kg [28]; 10 mg/100 g [41]. Juice + gradient: 50 mg/kg [59].	38 mg protein/kg fresh root [46].
Purity	34.1% [32].	63.6% [14].	ExoQuick 59.7% [32].	Not reported.	Not reported.	UC + ExoQuick + gradient: 83.3% [32].	Not reported.
Vesicle Integrity	EV type: spherical double-layer [33]; ACDV type: mostly spherical/cup-shaped intact bilayer. Horizontal: GrEVs are closed spherical structures <100 nm (cryo-EM) [35].	Spherical or cup-shaped vesicles with an intact bilayer [14,16].	Spherical/cup-shaped vesicles and intact bilayers [31,32,33].	The structure is cup-/disc-shaped or ellipsoidal, with an intact bilayer [29,30,31,34,35,36].	Spherical/cup-shaped vesicles with an intact bilayer are observed in the study [36,37].	The structures are cup-shaped, disc-shaped, or ellipsoidal, with an intact bilayer [24,25,26,31,35].	Spherical/cup-shaped vesicles with an intact bilayer are observed in the study [46].
Vesicle Stability	Buffer homogenization: colloidal stability (24 h, PDI < 0.25) [51]. Blender/grinding: stable after 3 freeze-thaw cycles [57]; freeze-dried at RT for 60 days [24]. Squeezing: at 4 °C for 2 weeks [58]. Horizontal: 147.2 ± 2.6 nm after exposure to gastric (pH 2.0) and intestinal (pH 6.5) fluids [16].	It offers continuous operation and is easily scalable.	High stability was reported, indicating that the combination method improved stability [32].	Not reported.	Not reported.	Freeze-dried at room temperature (RT) for 60 days [24,26]. subjected to 3 freeze-thaw cycles [57]; stored at 4 °C for 7 days [27,36,45]; exposed to simulated gastrointestinal (GI) conditions for 60 min [28]; and evaluated in vivo for circulation [40].	Stable at 4 °C for 7 days; lyophilization intact [46].
Cost and Scalability	High cost associated with small-batch research.	Continuous operation; easily scalable. Combined with	Simple and eco-friendly; large-scale.	Moderate cost; membrane throughput is limited.	It is simple and easily scalable.	Good morphology, but hard to scale.	Expensive consumables and small loading capacity make it hard to scale.
Applications and Limitations	Mainstream isolation often requires further purification.	dUC/gradient to improve purity; pilot-scale scale-up and complex optimization.	Simple with a higher yield than dUC, but residual polymer interferes.	Simple high-yield crude; low purity; cannot distinguish between same-size impurities.	Decontamination/purification after gradient: pure and intact, with limited capacity.	Usually occurs after dUC and is time-consuming.	After dUC/UF, there is high integrity and efficient free-protein removal, but only small-batch, high-purity yields are achieved.

### 2.2. Physicochemical Characterization

The physicochemical properties of ginseng exosome-like nanovesicles directly influence their biodistribution, cellular uptake, and therapeutic efficacy. Nanoparticle tracking analysis (NTA) is the most commonly used technique for particle size measurement, calculating size distribution and concentration by real-time tracking of Brownian motion. A study utilized tangential flow filtration systems to isolate ginseng exosomes, and NTA measurements revealed an average diameter of 159.5 nm [14]. Transmission electron microscopy (TEM) provides high-resolution morphological images; vesicles typically display a characteristic “saucer-shaped” or spherical structure with a discernible phospholipid bilayer. Dynamic light scattering (DLS) offers rapid assessment of overall sample homogeneity but should be used complementarily with NTA to ensure data reliability. Cryo-electron microscopy (Cryo-EM), the gold standard for structural integrity assessment, visualizes vesicle morphology under near-physiological conditions and accurately distinguishes intact vesicles from ruptured membrane fragments [21]. Furthermore, nanovesicles of different sizes exhibit notable differences in compositional profile and cellular uptake efficiency [61]; researchers have also developed a copper superparticle-based Bragg scattering coupled luminescence strategy for sensitive detection of ginseng exosomal miRNA [62].

Zeta potential is an important parameter for evaluating colloidal stability. The surface of ginseng exosome-like nanovesicles typically carries a negative charge, with Zeta potentials generally ranging from −15 mV to 30 mV, attributable primarily to phosphate groups of phospholipids and surface-adsorbed acidic glycoproteins. A high absolute zeta potential provides sufficient electrostatic repulsion to prevent vesicle aggregation. Stability experiments indicate [15] that particle size and Zeta potential remain relatively stable during short-term storage at 4 °C (72 h), whereas repeated freeze–thaw cycles disrupt the lipid bilayer and cause cargo leakage.

We incorporate characterization measurements for PG-EVLPs, including particle concentration, size distribution, morphology, membrane integrity, protein markers, lipid composition, RNA cargo, small-molecule/ginsenoside profiles, purity assessment against protein and non-vesicular contaminants, as well as particle-to-protein ratio and other relevant purity metrics in Table 2.

## 3. Active Compound Composition

### 3.1. Nucleic Acid

Nucleic-acid cargo contained within ginseng-derived nanovesicles has been hypothesized to contribute to their biological effects; therefore, a critical collation of the available experimental evidence would summarize the current research status and outstanding limitations.

High-throughput sequencing analyses have suggested that ginseng exosome-like nanovesicles are enriched in diverse microRNAs (miRNAs), among which *miR-159*, *miR-166*, and *miR-396* appear to be highly conserved in ginseng [64]. Using small-RNA-sequencing approaches, Lee et al. profiled gEV cargo and detected 87–398 distinct miRNA species in ginseng roots collected across different years, with *pgi-miR6136a.2* reportedly showing higher relative abundance in vesicle fractions than in crude ginseng extracts [46]; Xu et al. and Tan et al. performed recipient-cell RNA-seq and detected plant-origin miRNA reads in stem cells and endothelial cells after vesicle incubation [30,65]. For targeted nucleic-acid quantification and in situ visualization, Ren et al. used RT-qPCR to measure *miR-2118* in PG-EVLPs-treated chondrocytes [49], while Wang et al. combined FISH with qPCR and detected *pgi-miR6135j* signals in macrophage cultures and in joint tissues of CIA-model mice [60]. For in vitro biosensing quantification of isolated vesicle-derived miRNAs, Lyu et al. reported two electrochemiluminescence platforms: a copper superparticle-based strategy for detecting ginseng exosomal miRNAs [62], and a Zn_2_GeO_4_:Mn luminescent superparticle/Au–Ag bimetallic nanoarray platform for sensitive detection of ginseng-derived *miR-156a* [63].

Fluorescence-based confocal imaging has been widely adopted to characterize cellular internalization of ginseng-derived vesicles. Tan et al. recorded uptake of CM-DiI-labeled GExos by HUVEC endothelial cells, and Ren et al. observed internalization of PKH26-labeled PG-EVLPs in chondrocytes [30,49]. Wang et al. detected DiL-labeled GDNP-associated fluorescence in the joint tissue of orally dosed CIA mice, and Lee et al. visualized uptake of FITC-labeled gEVs by breast and colon tumor cell lines [46]. To validate physical interactions between plant-origin miRNAs and mammalian mRNA 3′-UTR targets, dual-luciferase reporter assays were employed. Ren et al. constructed wild-type and seed-mutant 3′-UTR reporter plasmids and obtained data suggesting a possible interaction between *pgi-miR-2118* and the *SLC39A7* 3′-UTR, whereas Wang et al. combined reporter assays with FISH co-localization and provided suggestive evidence for *pgi-miR6135j* binding to the *KRAS* 3′-UTR [49,60]. Nevertheless, most other candidate miRNA–target relationships are derived solely from bioinformatic predictions and have not yet been experimentally validated.

Cell-based gain- and loss-of-function assays provide cellular-level evidence for the potential bioactivity of plant-derived miRNAs. Ren et al. treated chondrocytes with synthetic *miR-2118* mimics and recapitulated PG-EVLPs-mediated anti-ferroptotic phenotypes; co-treatment with a *miR-2118* inhibitor attenuated these cytoprotective effects in vitro [49]. Wang et al. employed miRNA mimics, inhibitors, and RNase-pre-digested vesicle controls to dissect the functional contribution of miRNA: in macrophage cultures, *pgi-miR6135j* mimics appeared to recapitulate the biological responses and corresponding inhibitors abrogated GDNP-triggered inflammatory signaling changes; in CIA mice, disease progression was compared between animals receiving intact PG-EVLPs and RNase-pre-digested PG-EVLPs lacking intact vesicle-encapsulated RNA [60]. These observations suggest that miRNA may contribute to the biological activity of PG-EVLPs. Lee et al. performed stand-alone transfection experiments, delivering synthetic *pgi-miR6136a.2*/*Seq46-1* miRNA mimics into breast- and colon-derived tumor cell lines in the absence of native gEV particles; subsequent proliferation and migration assays revealed apparent anti-proliferative and anti-migratory phenotypes induced by these synthetic miRNA sequences [46]. These outcomes reflect the activity of exogenously introduced synthetic miRNAs and cannot be directly extrapolated to the function of these sequences when naturally packaged within native gEV particles. Sequencing has also detected mRNA and lncRNA fragments inside ginseng-derived nanovesicles, but functional data for these non-miRNA nucleic-acid components remain absent.

Collectively, the available evidence raises the possibility that nucleic-acid cargo within ginseng-derived nanovesicles may be involved in multiple cellular regulatory events, although direct causality has been established in only a limited number of studies. We have collated the reported findings on nucleic-acid detection, vesicle internalization, target-binding verification, and cell-level functional assays (Table 3).

### 3.2. Proteins

Western blotting and super-resolution imaging have been used to validate the protein markers of ginseng-derived nanovesicles. CD63, CD9, and TSG101 were detected by western blot in ginseng root-derived exosomes [19], and CD63 and CD81 were confirmed in ginseng-derived exosomes from root cell suspension culture [33]. The plant-specific marker PEN1 and ubiquitin were both detected in PNVs and PMVs [55] Zhang et al. verified the positive EV marker TET8 while excluding the cytoplasmic marker actin, the chloroplast marker RbcL, and the nuclear marker histone H3, thereby confirming vesicle purity [36]; similarly, TET-8 expression was elevated by 147.2% in TFF-purified vesicles [14].

Proteomic profiling has been applied to characterize the protein cargo. Cao et al. identified 3129 proteins in PG-EVLPs and classified them into three Gene Ontology categories [40]; GO and KEGG annotation of PG-EVLPs proteomes was also performed, and 86 GEN proteins were found to be homologous to human proteins [25]. Red ginseng RGNVs and ginseng GNVs were additionally profiled by Orbitrap-based proteomics and LC-MS/MS, respectively [16,66].

Functional attribution experiments have linked vesicle proteins to bioactivity. Digestion of PG-EVLPs with proteinase K abolished the induction of M1-polarization markers in macrophages, indicating that PG-EVLPs proteins may participate in the immunomodulatory effect of these particles. In osteogenic cultures, heat inactivation of RGNVs at 100 °C for 30 min only partially reduced mineral deposition, suggesting that heat-labile protein components contribute to osteogenic activity together with heat-stable components [66].

### 3.3. Lipids

Lipidomic analyses indicate that the vesicle membrane is composed primarily of phosphatidylcholine (PC), phosphatidylethanolamine (PE), sphingomyelin (SM), and small amounts of cholesterol, along with distinctive plant sphingolipids (e.g., phytosphingosine) that confer membrane properties distinguishing them from animal-derived exosomes. The high PC and PE content imparts membrane flexibility and fluidity, facilitating fusion with target cell membranes. The glycosyl moieties of ginsenosides (Rb1, Rg1, Re, etc.) embedded in the membrane form hydrogen bonds with lipid headgroup regions, altering local membrane curvature and permeability and enhancing vesicle bioavailability [67,68,69,70]. Rg1 and Re, for instance, promote vesicle fusion with bone cells by modulating membrane fluidity, activating BMP2/4 and p38 phosphorylation signaling pathways to stimulate osteogenic differentiation [55].

Experimental evidence links vesicle lipids to delivery function. Kim et al. extracted PG-EVLPs lipids using the Bligh-Dyer protocol and used the extracted lipid content as a transfection reagent to deliver exogenous miRNAs; delivery of *ptc-miR396f* via ginseng-derived lipids produced the strongest gene-silencing effect, reducing *c-MYC* and *BCL2* expression by 58% and 76%, respectively, and DiD-labeled PG-EVLPs showed superior cellular uptake compared with DiD-labeled artificial liposomes [25]. Cao et al. further suggested that ceramide lipids and proteins of PG-EVLPs may play an important role in macrophage polarization via TLR4 activation, although this interpretation remains to be experimentally confirmed [40].

### 3.4. Ginsenosides

At present, ginsenosides’ pharmacological effects are validated by purified free ginsenosides, conventional ginseng crude extracts, isolated native PG-EVLPs, and engineered PG-EVLPs loaded with additional exogenous therapeutic cargos. Purified free ginsenosides exhibit well-documented anti-inflammatory, anti-osteoporotic and antioxidant activities in vitro, yet they suffer from poor oral bioavailability and rapid gastrointestinal degradation [71]. Conventional ginseng crude extracts reproduce multiple in vivo therapeutic phenotypes, but these complex mixtures contain various molecular components, which makes it impossible to be fully recapitulated by individual ginsenosides owing to combinatorial effects among multiple extract constituents [72]. As confirmed by liquid chromatography tandem mass spectrometry, both the interior and membrane surface of PG-EVLPs harbor abundant protopanaxadiol type (Rb1, Rd) and protopanaxatriol type (Rg1, Re) ginsenosides, with certain ginsenoside species showing enrichment within nanovesicle fractions [73]. Engineered ginseng-derived nanovesicles were constructed by encapsulating a mitochondrial-directed triptolide prodrug and modifying the vesicle surface with MCSP targeting scFv. This engineered PG-EVLPs exhibited improved tumor targeting and synergistic therapeutic effects to remodel the immunosuppressive tumor microenvironment [74]. A recent study investigating native PG-EVLPs demonstrated bioactivity that cannot be fully recapitulated by free ginsenosides alone. When macrophages were treated with individual free ginsenosides (Rb1, Rg1, Rg3) at concentrations equivalent to their intrinsic levels within 10 μg/mL PG-EVLPs, Rb1 and Rg1 scarcely suppressed *STT3A* transcription, while Rg3 yielded only moderate inhibition (~44.5%). In contrast, intact GDEs elicited 96% downregulation of *STT3A* [57]. These observations indicate that robust *STT3A* repression cannot be attributed solely to these three ginsenosides. Instead, the transcriptional suppression of *STT3A* likely arises from combinatorial effects of multiple PG-EVLPs components, including lipids and major and minor ginsenosides.

Seo et al. determined the intrinsic ginsenoside composition of ginseng-derived nanovesicles (GDNs) by LC-MS (Rb1 64.5%, Rg1 31.1%) and compared their anti-osteoclastogenic activity with that of free Rb1, free Rg1, and Rb1:Rg1 mixtures (7:3 and 5:5 ratios) by TRAP staining [1]. Although the mixture ratios were based on the vesicle-intrinsic composition, the concentrations of free ginsenosides were selected according to literature-reported [2] effective concentrations (Rb1 at 1 µg/mL; Rg1 at 100 µg/mL) rather than being converted to concentrations matching the ginsenoside load of the GDN treatment (1 µg/mL); GDNs nonetheless suppressed osteoclast differentiation more effectively than any single ginsenoside or mixture [15]. In a more rigorous content-matched design, Choi et al. quantified ginsenoside contents within ginseng root-derived exosome-like nanoparticles (GrDENs, 2 × 10^9^ particles/mL) by HPLC-MS and calculated the mass concentration of each major ginsenoside (Re 3.8, Rg1 3.5, Rb1 1.1, and Rc 0.7 µg/mL), so that free Re, Rg1, Rb1, and Rc were administered at concentrations consistent with those determined within GrDENs. In H_2_O_2_-exposed keratinocytes, free Re alone significantly suppressed MMP2 and MMP3 mRNA expression, and a mixture of the four ginsenosides slightly further enhanced MMP3 inhibition; importantly, intact GrDENs showed stronger suppression than any individual ginsenoside group, implying that other minor ginsenosides or vesicular components may contribute to the inhibitory activity [56].

Small amounts of ginseng polysaccharides are also present, likely participating in immune regulation and gut microbiota modulation as glycoprotein complexes [2]. Ginseng polysaccharides can upregulate the relative abundance of short-chain fatty acid-producing bacterial genera, promoting the generation of metabolites, such as butyrate, which indirectly suppress tumor growth, and the synergistic action of ginsenosides and polysaccharides. The former directly targets cellular signaling pathways (e.g., inhibiting Smad2/3/Snail and PI3K/Akt pathways), and the latter acts indirectly through gut microbiota modulation, which is a main reason why vesicles outperform individual saponins in efficacy [5,75]. Although ginseng polysaccharides are well-recognized pharmacological constituents of *Panax ginseng*, no study to date has performed qualitative or quantitative characterization of polysaccharides within purified ginseng-derived nanovesicles, representing a knowledge gap that warrants future investigation.

Five major categories of bioactive substances loaded in PG-EVLPs are summarized in Table 3, including nucleic acids, functional proteins, membrane lipids, ginsenosides, and polysaccharides. These ingredients jointly produce synergistic therapeutic effects via cross-kingdom regulation. At present, the loading mechanism of ginsenosides embedded in lipid bilayers remains unclear, and comparative analysis of active components under different extraction methods is still insufficient. It should be emphasized that no study has tested whether PG-EVLPs activity is retained after selective depletion of ginsenosides and polysaccharides inside.

## 4. Pharmacological Activities

### 4.1. Anti-Inflammatory and Immunomodulatory Effects

PG-EVLPs show significant biological activity in anti-inflammatory and immunomodulatory contexts. Ginseng-derived exosome-like nanoparticles attenuated LPS-induced inflammatory responses in macrophages by reducing toll-like receptor 4 (TLR4) glycosylation, thereby inhibiting LPS binding; this suppression led to decreased production of nitric oxide and proinflammatory cytokines (IL-1β, IL-6, TNF-α), as well as inhibition of intracellular ROS accumulation and NF-κB activation, and markedly improved survival in an LPS-induced sepsis mouse model [17]. The vesicles also activate autophagy and target the IKK/IκB/NF-κB pathway, promoting the transition of macrophages from the pro-inflammatory M1 phenotype to the anti-inflammatory M2 phenotype, thereby alleviating inflammatory responses [44]. The adaptive regulation of immune function by ginseng contributes to its overall pharmacological efficacy [76].

In inflammatory bowel disease (IBD) models, vesicles significantly attenuate intestinal inflammation and protect the mucosal barrier through coordinated regulation of TLR4/MAPK and p62/Nrf2/Keap1 pathways [28]. Following oral administration, the vesicles exhibit a retention effect in the intestinal tract lasting up to 48 h, remodeling the gut microecology by increasing the abundance of beneficial bacteria such as Lactobacillus and Bifidobacterium, suppressing pro-inflammatory Th17 cell differentiation, and promoting regulatory T cell (Treg) expansion—collectively restoring intestinal immune homeostasis [17]. Ginsenoside Rg2 carried by the vesicles regulates the NF-κB/NLRP3 signaling pathway, markedly inhibiting caspase-1 activation and the maturation and release of pro-inflammatory factors including IL-1β and IL-18 [77]. Ginsenoside Compound K ameliorates DSS-induced IBD by modulating tryptophan metabolism and activating the aryl hydrocarbon receptor [78]; network pharmacology studies have further uncovered the multi-target mechanisms underlying ginseng’s therapeutic effects on ulcerative colitis [79]. The potential of ginsenosides as therapeutic candidates for IBD has been systematically evaluated [80], with anti-inflammatory actions mediated through gut microbiota regulation and intestinal barrier repair. Additionally, miRNAs within the vesicles ameliorate rheumatoid arthritis symptoms by mediating *KRAS*-MAPK signaling [60] and inhibit adipocyte differentiation through AMPK-mediated pathways, revealing a novel metabolic regulatory function [14].

Regarding innate immune regulation, the vesicles are efficiently internalized by macrophages and dendritic cells (DCs) via clathrin-mediated endocytosis, activating the TLR4/MyD88-dependent pathway and promoting secretion of key cytokines such as IFN-β and IL-12, thereby enhancing the initial antiviral and antibacterial immune responses. In adaptive immunity, the vesicles promote B cell proliferation and differentiation, enhance IgA secretion, and maintain the integrity of the intestinal mucosal immune barrier; concurrently, they deliver *miR-166* to suppress Th2-type cytokine (IL-4, IL-13) expression, attenuating allergic airway inflammation [57]. Engineered ginseng-root-derived exosome-like nanovesicles can be further functionalized for pulmonary inflammatory disease treatment. For instance, neutrophil membrane-camouflaged PG-EVLPs loaded with *miR-182-5p* can target the *NOX4*/*Drp-1*/NLRP3 axis and mitigate inflammatory damage in sepsis-triggered acute lung injury in vivo [15]. This “intelligent” bidirectional immunomodulatory property, capable of both enhancing anti-infective immune responses and suppressing excessive immune activation, imparts distinct advantages to the vesicles in treating complex immune-related disorders.

### 4.2. Antitumor Activity

Ginseng exosome-like nanovesicles exhibit multi-target, multi-pathway antitumor activity. The vesicles inhibit the viability of various tumor cell lines in a dose-dependent manner. In hepatocellular carcinoma HepG2 and lung cancer A549 cells, the mechanism involves induction of G0/G1 cell cycle arrest, upregulation of the pro-apoptotic Bax to anti-apoptotic Bcl-2 ratio, and activation of the caspase-3-dependent apoptotic pathway. *miR-159*, enriched in the vesicles, targets and suppresses epidermal growth factor receptor (EGFR) and its downstream PI3K/AKT signaling axis, disrupting growth signal transduction in tumor cells [81]. The vesicles also modulate PD-L1 expression, inhibiting immune evasion in non-small cell lung cancer (NSCLC), enhancing T cell-mediated cytotoxicity, and promoting IL-2 and IFN-γ secretion [33]. Ginsenoside Rh2 carried by the vesicles enhances chemotherapy sensitivity in NSCLC cells through regulation of the *USP22*-*RRM2* axis [50].

In the context of tumor immune microenvironment modulation, PG-EVLPs reprogrammed tumor-associated macrophages (TAMs) to increase CCL5 and CXCL9 secretion for recruiting CD8^+^ T cells into the tumor bed, which synergized with PD-1 monoclonal antibody therapy without detected systemic toxicity [81]. In a B16F10 melanoma model, PG-EVLPs promoted the polarization of M2 to M1 macrophages in a TLR4/MyD88-dependent manner, and the resulting ROS production increased apoptosis of melanoma cells and suppressed tumor growth [40]. HSP70 on the vesicle surface can act as a danger signal, activating DC maturation and antigen presentation to initiate broader antitumor immune responses [25]. Edible ginseng-derived exosomes serving as drug delivery vehicles can reduce the required dose of chemotherapeutic agents (e.g., cisplatin) while improving antitumor efficacy [37]. When combined with conventional chemotherapeutic drugs, the vesicles not only reduce systemic toxicity but also enhance antitumor effects through synergistic mechanisms.

### 4.3. Tissue Repair and Neuroprotection

In cutaneous wound healing, the vesicles activate PI3K/AKT and MAPK/ERK signaling pathways to promote keratinocyte proliferation and migration, while simultaneously stimulating fibroblast collagen synthesis via the TGF-β/SMAD pathway [22]. Studies show that vesicle treatment enhances the proliferative capacity of HaCaT keratinocytes, BJ fibroblasts, and HUVECs, promoting angiogenesis and cell migration [20]. In diabetic wound models, the vesicles act on PI3K/AKT/HIF-1α and TLR4/MyD88/MAPK pathways to promote neovascularization, alleviate oxidative stress, and modulate macrophage polarization, ultimately accelerating wound closure. Ginsenoside Rb1 exerts dual therapeutic mechanisms in pressure injury repair through SIRT1-AMPK-mediated ferroptosis inhibition and angiogenesis activation [53].

Regarding organ protection, ginseng exosome-like nanovesicles exhibit broad cardioprotective effects. The vesicles confer significant protection against myocardial ischemia/reperfusion injury by inhibiting oxidative stress and apoptosis [56]. The adaptive regulation of cardiovascular function by ginseng provides a pharmacological basis for its cardioprotective effects [82]. In hepatic protection, oral PG-EVLPs protected against liver injury and fibrosis in MASLD and ALD mouse models by restoring intestinal barrier integrity, rebalancing the gut microbiome, and downregulating the fibrosis-associated TIMP2 pathway in hepatic stellate cells [16]. In osteoporosis models, the vesicles demonstrate therapeutic potential through enhanced osteoblast activity [55].

In the domain of anti-aging and neuroprotection, ginseng has pharmacological value in the prevention and treatment of aging-related disorders [83]. The vesicles reduce oxidative stress levels in the hippocampus of D-galactose-induced aging mice, upregulate SOD and GSH-Px activity, and improve cognitive function. Research suggests that PG-EVLPs in-vitro assays demonstrated that antioxidant and anti-apoptotic bioactivities shift microglia toward an anti-inflammatory M2 phenotype. Intravitreal PG-EVLPs suppressed retinal neuroinflammation and enhanced retinal ganglion cell survival in a chronic ocular hypertension rat model, indicating its neuroprotective potential [43].

This section summarized the multi-target therapeutic effects of PG-EVLPs against inflammation, tumors, tissue injury, and degenerative diseases, with corresponding disease models and signaling pathways listed in Table 4. PG-EVLPs achieve bidirectional immune regulation and multi-pathway intervention through their endogenous ginseng-derived active compounds. However, most pharmacological studies remain at the cell model and animal model stage and are still lacking in humans. Plant-derived exosome-like nanovesicles from diverse medicinal plants, including turmeric, ginseng, ginger, garlic, grape, yam, tomato and grapefruit, share overlapping anti-inflammatory and immunomodulatory phenotypes, and their bioactivities are largely dictated by plant-specific secondary metabolites derived from their parent organisms [84]. Although PG-EVLPs possess a distinct cargo profile enriched in ginsenosides, direct head-to-head functional comparisons with other plant-derived exosome-like nanovesicles remain absent. Therefore, it cannot be determined which pharmacological phenotypes represent unique features specific to PG-EVLPs.

## 5. Delivery Applications

### 5.1. Natural Nanocarrier Delivery

As natural nanocarriers, ginseng exosome-like nanovesicles possess inherent biocompatibility and low immunogenicity, enabling them to evade clearance by the mononuclear phagocyte system [89]. The vesicles can serve as natural carriers for diverse therapeutic cargos—including small-molecule drugs, nucleic acids, and proteins—achieving efficient intracellular delivery [90]. Edible ginseng-derived exosomes functioning as drug delivery vehicles can reduce the effective dose of chemotherapeutic agents (e.g., cisplatin) while improving antitumor outcomes [37]. miRNAs within the vesicles can regulate recipient cell gene expression through cross-kingdom delivery mechanisms, demonstrating the potential of these vesicles as natural gene therapy carriers [64]. Following oral administration, the vesicles exhibit pronounced gastrointestinal retention, with a fraction entering the systemic circulation via the intestinal lymphatic system and reaching organs such as the liver and spleen. Ginseng-based nanoformulations also show delivery potential in other domains; for instance, ginseng nanoemulsion ameliorates male infertility by modulating sex hormones and oxidative stress [91].

### 5.2. Engineering Modification and Targeted Delivery

Researchers have pursued multiple strategies to enhance the targeted delivery capabilities of the vesicles. Neutrophil membrane-engineered ginseng root-derived exosomes loaded with miRNA 182-5p alleviated acute lung injury in sepsis by targeting the *NOX4*/*Drp-1*/NLRP3 signaling pathway [19]. Endothelium-targeted (VHPKQHR peptide-modified) ginseng-derived exosomes delivering IL-6 siRNA ameliorated hepatic ischemia-reperfusion injury by reducing inflammatory cytokine release, alleviating oxidative stress, restoring mitochondrial membrane potential, and driving macrophage M2 polarization [27]. M1 macrophage-targeted engineered extracellular vesicles derived from ginseng stems and leaves have been applied in targeted rheumatoid arthritis therapy [36].

### 5.3. Hydrogel Composite Delivery Systems

Hydrogel composite systems are promising platforms to achieve sustained local release of PG-EVLPs. A thermosensitive injectable hydrogel loaded with native PG-EVLPs can conform to periodontal pocket anatomy, inhibit periodontal pathogenic biofilm formation, promote M2 macrophage polarization and facilitate alveolar bone regeneration in periodontitis animal models [41]. In addition, pH/ROS dual-responsive hyaluronic acid-based hydrogel loaded with PG-EVLPs shows synergistic anti-bacterial, anti-inflammatory and angiogenic effects and accelerates healing of diabetic oral ulcer lesions in rats [38]. A multifunctional DNA hydrogel combining light-triggered gas therapy with controlled ginseng exosome release demonstrates significant efficacy in infected wound treatment [54].

This section covered three delivery forms of PG-EVLPs: natural oral carriers, cell membrane-engineered targeted vesicles, and composite hydrogel systems. Hydrogel platforms provide sustained local release, while modified vesicles enable organ-specific targeting. Current delivery strategies remain at the laboratory stage, and how vesicle modification schemes map to specific disease treatments requires further study.

## 6. Safety Evaluation and Clinical Translation Prospects

### 6.1. Safety and Biocompatibility

Studies on PG-EVLPs have become possible because several in vivo and in vitro studies have reported favorable biosafety for PG-EVLPs under their tested conditions. Hybrid PG-EVLPs did not trigger hemolysis at 100 µg/0.1 mL per mouse; vaccination with these particles produced no severe systemic inflammation and caused no detectable histological damage to major organs, including liver and spleen, with blood biochemical parameters remaining within normal physiological ranges [58]. In vitro cytotoxicity assays indicated that PG-EVLPs of the root extract preparations maintained more than 80% cell viability even at relatively 10 µg/mL concentrations [35]. Even after 72 h of treatment at high concentrations (30 μg/mL), GDVLNs showed no cytotoxicity to mouse macrophages [16,40], which complied with the ISO 10993-5 [92] cytotoxicity criteria for non-cytotoxic materials.

Sub-acute oral toxicity tests for PG-EVLPs demonstrated good in vivo tolerance up to 50 mg/kg/day; despite the liver being a primary accumulation organ for orally administered PG-EVLPs; no obvious hepatic or splenic histopathological lesions were observed, and serum hepatic renal biomarkers showed no significant deviation from vehicle controls. In intraperitoneal and intravenous injection of PG-EVLPs, no statistically significant abnormalities in hematological indices, liver and kidney biochemical markers, or organ histology (liver, spleen included) were recorded in treated mice compared with control animals [58].

PG-EVLPs, as an emerging natural nanodelivery system, require thorough safety evaluation and biocompatibility assessment as important prerequisites for their transition from basic research to clinical application. Available preclinical studies have demonstrated that PG-EVLPs exhibit favorable in vitro and in vivo biocompatibility and short-term safety, without obvious hemolysis, cytotoxicity, or organ damage, while the long-term safety profile remains insufficiently characterized.

### 6.2. Clinical Translation Challenges

To date, human evidence for ginseng-derived nanovesicles remains confined to three small studies, all addressing topical applications to the skin or vulvar mucosa rather than systemic or internal-organ indications. In the largest and the only controlled study, Wang et al. conducted a pilot human study enrolling 30 volunteers (mean age, 47 years) and reported that one month of microneedle-roller-assisted delivery of proanthocyanidin-loaded ginseng exosomes (Pro@Gin-Exo) significantly improved skin smoothness, elasticity, hydration, wrinkle depth, pigmentation, and pore size compared with the control group [51]. In a single-arm preliminary pilot observational study, Park et al. treated 14 women (mean age, 48.6 years; range, 24–63 years) with three sessions of dermaroller-assisted topical *Panax ginseng* exosomes combined with an adjunctive inner gel over a 6-week period; the total Vulvovaginal Symptom Questionnaire (VSQ) score decreased by a mean of −2.86 (95% CI, −4.76 to −0.95; *p* = 0.00648) and the total Day-to-Day Impact of Vaginal Aging (DIVA) score decreased by −5.43 (95% CI, −9.55 to −1.30; *p* = 0.0138), with significant improvement in the daily-activities domain, whereas the emotion and sexual-life domains showed only directional decreases that did not reach statistical significance; procedure-related pain was transient and subsided within 30 min [88]. At the case-report level, Wan et al. described two patients (a 32-year-old woman and a 37-year-old man) with postinflammatory hyperpigmentation following acne who underwent three sessions of microneedling therapy combined with topical application of a commercially marketed ginseng exosome formulation (P198 RECORE SB PLUS); the PIH severity scale improved from grade 2 (mild) to grade 1 (trace) in both patients as evaluated by two dermatologists, with no adverse effects reported [87].

Despite these encouraging signals, the existing human evidence shares critical limitations that preclude any conclusion on clinical efficacy. First, all three studies were small (*n* = 30, 14, and 2, respectively), and only the study of Wang et al. included a control group, whereas the other two were uncontrolled single-arm or case-level observations. Second, the tested products were not biochemically validated and purified PG-EVLPs preparations: the case report of Wan et al. used an off-the-shelf cosmetic formulation rather than a characterized vesicle product [87], and the experimentally fabricated preparations of the other two studies have not been subjected to batch-to-batch quality assessment [51,88]. Third, all reported endpoints were subjective or patient-reported (questionnaire scores and photographic grading) with short-term follow-up (2–6 weeks), and none of the studies assessed systemic exposure, pharmacokinetics, or long-term safety [51,87,88]. Notably, no clinical study has yet evaluated purified PG-EVLPs for systemic or internal-organ diseases, and no formally registered randomized controlled trial of PG-EVLPs has been published.

Clinical investigations on standardized *Panax ginseng* extracts have demonstrated measurable biological responses in human participants under both acute and sub-chronic supplementation regimens, although therapeutic outcomes remain inconsistent across trial populations. Standardized aqueous alcoholic ginseng root extracts, characterized by major ginsenosides, including Rg1, Re, Rb1, Rc, Rb2, and Rd, have shown significant modulatory effects on postprandial glucose homeostasis in healthy middle-aged adults; pre-meal administration of ginseng extract effectively reduced postprandial glucose incremental area under the curve and peak glucose concentration in randomized crossover trials, indicating acute metabolic regulatory capacity of purified ginseng extract preparations [93]. Beyond metabolic regulation, double-blind placebo-controlled trials reported that *Panax ginseng* extract intervention modulated stress-related physiological indicators and brain functional connectivity in subjects with self-reported stress symptoms [94]. Nevertheless, considerable inter-study heterogeneity exists regarding extract dosage, ginsenoside standardization, intervention duration and participant baseline status [95], which partially accounts for conflicting efficacy observations among different clinical studies, highlighting the necessity for well-standardized extract materials in future human trials.

Various oral *Panax ginseng* preparations, including root powder-filled capsules and composite herbal formulations, have been evaluated in phase relevant human clinical trials targeting chronic respiratory disease, fatigue and cognitive function, providing translational clinical evidence for whole-material ginseng products beyond purified extracts. G115-based ginseng capsule preparation, one of the most frequently tested ginseng formulations, was assessed in a large-scale randomized controlled trial enrolling patients with moderate to very severe chronic obstructive pulmonary disease; while ginseng capsule treatment exhibited a good safety profile with adverse-event rates comparable to placebo, no statistically significant reduction in disease exacerbation frequency was observed after long-term oral administration, implying limited direct disease-modifying efficacy for this patient cohort under the tested dosage regimen [96]. Pilot clinical studies further indicated that conventional white ginseng powder capsule preparations could partially alleviate subjective stress-related discomfort in fatigued healthy volunteers, yet cognitive-function improvement was less prominent compared with highly standardized extract counterparts, suggesting that formulation type and raw material processing status jointly determine clinical readouts of ginseng preparations [97]. Compared with well-characterized ginseng extracts, most whole-root ginseng preparations lack unified ginsenoside content specifications, and inter-batch variation of raw botanical materials constitutes a major confounding factor for interpreting clinical outcomes across independent human trials [98].

Standardization of isolation and purification constitutes the main bottleneck impeding industrialization. Methodological and quality control discrepancies across laboratories make batch-to-batch consistency difficult to ensure [9]. Large-scale production processes have yet to be established, and scaling from laboratory-grade milligram quantities to industrial-grade gram yields presents a series of engineering challenges [99]. Although promising preclinical data have demonstrated anti-inflammatory and immunomodulatory effects of PG-EVLPs, formally registered clinical trials remain absent to date, and only limited case report evidence in human skin application is available. Further efforts in standard production, quality control and pharmacokinetic evaluation are required before advancing to formal clinical investigation.

## 7. Conclusions

*Panax ginseng*-derived EV-like particles contain inherent bioactive components including various ginsenosides. These endogenous bioactive substances confer intrinsic therapeutic effects on the nanovesicles. Collectively, the currently available evidence is primarily preclinical and largely relates to short-term tolerability. However, the long-term safety profile of PG-EVLPs remains insufficiently characterized. In particular, chronic toxicity, potential immunogenicity, systemic biodistribution, and long-term tissue accumulation of PG-EVLPs have not yet been systematically evaluated and warrant further investigation.

Several challenges remain for PG-EVLPs research. Extraction and purification workflows require further optimization, and researchers have not established reliable, scalable production. PG-EVLPs research should focus on concrete technical priorities such as standardized botanical-material sources and cultivation conditions, harmonized isolation protocols, orthogonal characterization workflows, quantitative cargo profiling, rigorous quality control systems, development of functional potency assays, validation of underlying mechanisms of action, comprehensive pharmacokinetic and biodistribution assessments, and systematic evaluation of chronic toxicity and immunogenicity risks. Researchers still need to conduct further investigations to advance this research field.

## Figures and Tables

**Table 2:** Physicochemical characterization.

Characterization Dimension	Recommended Technique(s)	Key Reported Parameters	*Panax Ginseng*-Derived EV-like Particles Physicochemical Characterization (PG-EVLPs)	Recommendations
Particle Size	Nanoparticle tracking analysis (NTA), dynamic light scattering (DLS), transmission electron microscopy (TEM), cryo-electron microscopy (CryoEM).	Determine the mean/median diameter, size distribution (PDI), and particle size range.	PG-EVLPs overall ranges from 29 to 499 nm. Mostly EV type (culture supernatant extraction) at 50–150 nm [33,34,35]. Mostly ACDV type (buffer homogenization/blender/juice/squeeze/grind) at 100–250 nm [14,16,25,26,31,32,33,34,35,36,39,40,47,57].	Multi-method characterization is recommended for mutual validation, and vesicle size should not be determined using a single approach.
Particle Concentration	Nanoparticle tracking analysis (NTA); nano-flow cytometry (nFCM).	Particles per mL.	PG-EVLPs overall ranges from 10^6^ to 10^13^ particles/mL, varies with isolation [14,16,24,25,26,32,33,53]. TFF 3.8 × 10^6^ particles/mL vs. dUC 1.4 × 10^9^ particles/mL [16].	Apply refractive-index correction for sizing, use vesicle-specific fluorescence to filter out false positives, and optimize dilution to reduce coincidence.
Morphology	Transmission electron microscopy (TEM) with negative staining; cryo-electron microscopy (CryoEM).	Vesicle shape, bilayer visibility, presence of debris.	PG-EVLPs are cup-shaped, disc-shaped, or ellipsoidal-shaped lipid bilayer vesicles.	Minimize drying artifacts during negative staining. Optimize ice thickness for cryo-EM and acquire a sufficient number of micrographs from randomly selected areas.
Membrane Integrity	Cryo-EM.	Ratio of intact/ruptured vesicles; degree of cargo leakage.	Cryo-EM: closed spherical structure <100 nm, intact bilayer [35].	Same as above.
Surface Charge	Zeta potential measurement (DLS-based electrophoretic light scattering).	Zeta potential (mV).	PG-EVLPs negative zeta potential from −8.14 to −39.0 mV (mostly −15 to −30 mV) [15,20,24,25,32,36,39,40,47].	Control buffer pH and ionic strength; dilute samples appropriately to avoid interparticle interference.
Colloidal Stability	Zeta potential; serial time-point NTA/DLS.	Zeta-potential absolute value; change of size/PDI over incubation time.	Colloidal stability over 24 h with stable size/PDI (PDI < 0.25) [51]. Size/zeta/concentration unchanged after 3 freeze-thaw cycles [57]. Freeze-dried stable at RT for 60 days [24]. Stable at 4 °C for 2 weeks [58], 4 °C 7 days [27,36,45].	Zeta potential is affected by buffer pH, ionic strength and viscosity. Repeated sampling for serial measurements may induce particle aggregation.
Protein Marker Cargo	Western blot; LC-MS/MS proteomics.	Marker protein abundance, total vesicular protein content.	PG-EVLPs were confirmed to contain detectable vesicle marker proteins: plant-EV positive markers TET-8 [14,36] and PEN1 [55] negative contaminant markers for purity validation: cytoplasmic Actin, chloroplast RbcL, and nuclear Histone H3 [36].	Include vesicle-enriched proteins for identity and organellar contaminants for purity assessment; set appropriate experimental controls.
Lipid Composition	Lipidomics LC-MS/MS.	Lipid classes and relative abundance	PG-EVLPs were found to contain detectable phosphatidylcholine (PC), phosphatidylethanolamine (PE), sphingomyelin (SM), palmitic/palmitoleic acid, and trilinolein [25,28,30,31,35,41,56], and were mainly composed of PC [19,25].	Compare lipid profiles against source tissue; run extraction blanks and lipid standards to reduce background interference.
RNA Cargo	Small-RNA sequencing; qRT-PCR; capillary electrophoresis (Bioanalyzer).	RNA yield, miRNA profiles, RNA size distribution.	PG-EVLPs conserved plant miRNAs enriched: *miR-2118* [48,49]; *pgi-miR6135j* [60]; *miR-182-5p* [19,45]; *miR-396a-3p* [62]; *miR-156a* [63].	Use extraction blanks and spike-in controls; strictly prevent RNase contamination.
Small-Molecule Cargo	Untargeted or targeted LC-MS/MS; GC-MS.	Metabolite profiles, relative abundance.	Multiple secondary metabolites were detected in PG-EVLPs, either encapsulated within the lumen or associated with the membrane. Metabolomic profiling classified the exosomal cargo, and ginsenosides Rg1, Re, and Rb1 were quantified by HPLC, while UHPLC/IM-QTOF-MS revealed a diverse ginsenoside composition [30,31,34].	Employ reference standards; prepare matrix-matched controls and limit metabolite degradation.
Ginsenoside Cargo	LC-MS/MS; HPLC-UV.	Identity, ginsenoside concentrations.	PG-EVLPs contained ginsenosides (Rg1, Re, F2, Rb1, Rg3, Rc, Rh2, among others) detected by HPLC/LC-MS [14,15,26,31,47]. UHPLC/IM-QTOF-MS revealed seven ginsenosides common to all isolation methods [31] and quantified Rg1/Rg3/Rb1 [14,26].	Same as above.
Purity Metrics	Particle-to-total-protein ratio; TEM; western-blot for contaminant markers; LC-MS/MS.	Particle/μg total-protein; residual protein, lipoprotein, polymer contaminant level	Purity calculated from PG-EVLPs size distribution (50–150 nm): UC alone 34.1%, ExoQuick 59.7%, UC + ExoQuick + gradient 83.3% [32].	Evaluate purity by particle-to-total-protein ratio. Apply TEM for morphology, western blot and LC-MS/MS for contaminant marker detection.
Aggregation	NTA; DLS (PDI); TEM; Cryo-EM.	PDI value; appearance of large-size particle populations.	PG-EVLPs show low polydispersity: PDI 0.12–0.3 across preparations [15,17,24,25,35,39,51,60].	Compare PDI values and examine micrographs to confirm aggregation.

**Table 3:** Active compound composition of *panax ginseng*-derived EV-like particles.

Category	Nucleic Acid	Proteins	Lipids	Ginsenosides	Polysaccharides
Reported constituents in PG-EVLPs	*pgi-miR6135j* [60]; *miR-2118* [48,49]; *miR-182-5p* [19,45]; *miR-396a-3p* [62]; *miR-156a* [63].	Plant-EV markers: TET8 [14,36], PEN1 [55]; HSP70, HSP90, glycosyltransferases [25,41]. mammalian-related: CD63, CD81, TSG101 [19,33].	Phosphatidylcholine (PC), phosphatidylethanolamine (PE), sphingomyelin (SM), phytosphingosine, trace cholesterol, palmitic/palmitolinoleic acid, trilinolein [25,28,31,35,41].	Rg1, Re, Rb1, Rd, Rg3, Rh2, F2, Compound K, Rc [14,15,26,31,50,56,57].	Not reported
Location	Luminal interior, protected by lipid bilayer [25,60].	TET8: transmembrane protein [14,36]; HSP70/HSP90: luminal or surface-associated [25,35].	Major constituents of the vesicle lipid bilayer [25,35].	Intra-vesicle lumen and embedded within lipid bilayer; surface-adsorption risk [14,15].	Not reported
Tissue source	Fresh ginseng root, stem-leaf, cell-culture supernatant [14,33,34,35,36].	Fresh ginseng root, stem-leaf, cell-culture supernatant [14,35,36].	Fresh ginseng root, cell-culture supernatant [25,35].	Fresh ginseng root, stem-leaf [14,31].	Not reported
Biological source	*Panax ginseng C. A. Meyer.*	*Panax ginseng C. A. Meyer*.	*Panax ginseng C. A. Meyer*.	*Panax ginseng C. A. Meyer*; Red-ginseng processed material [66].	Not reported
Detection method	Small-RNA high-throughput sequencing; qRT-PCR; FISH combined with qPCR [44,48,49,59,60].	Western blot; LC-MS/MS-based proteomics [14,19,33,36].	LC-MS/MS lipidomics; untargeted lipidomic profiling [25,28,31,35,41].	UHPLC/IM-QTOF-MS; HPLC-UV; LC-MS/MS [14,15,26,31,47].	Not reported
Cargo expression in recipient cells	Recipient-cell RNA-seq detected plant-origin miRNA (let-7 family and the *miR-17-92* cluster) reads in endothelial cells after PG-EVLPs incubation [30]. Through qRT-PCR quantified *miR-2118* in PG-EVLPs-treated chondrocytes [49]. FISH detected *pgi-miR6135j* signals in macrophages and joint tissues [60].	Not reported	Not reported	Not reported	Not reported
Cellular uptake	Labeled-vesicle tracing, such as PKH26-, DiI/DiL-, DiO/DiO-, CM-DiI-, or DiD-labeled PG-EVLPs were efficiently internalized by diverse recipient cells—including chondrocytes (SW1353), macrophages (RAW264.7), HUVECs, and immune cells [17,27,30,40,49]. In addition, traced in vivo to joint tissue of CIA mice and the gastrointestinal tract after oral gavage by confocal microscopy, flow cytometry, and invivo imaging [25,28,60].	PKH26-labeled PG-EVLPs/PG-EVLPs -PH taken up by M1 macrophages (uptake-rate assay) [36] DiD-labeled PG-EVLPs tracked by in vivo imaging [25].	CM-DiI-labeled PG-EVLPs internalized by HUVECs [30]. DiD-labeled PG-EVLPs tracked by in vivo imaging [25].	Not reported	Not reported
Target binding	Dual-luciferase reporter: *pgi-miR6135j*-*KRAS* 3′-UTR [60]; *miR-2118-SLC39A7* 3′-UTR [48,49]; most other miRNA-target relationships derived only from bioinformatic prediction.		Not reported	Not reported	Not reported
Associated bioactivities	In the loss-of-function design, RNase predigestion of PG-EVLPs abolished their anti-inflammatory effects, directly excluding a role for vesicle proteins; in gain- and loss-of-function designs, synthetic miRNA mimics (*pgi-miR6135j, pgi-miR-2118*) recapitulated and miRNA inhibitors reversed the PG-EVLPs-mediated phenotypes, with dual-luciferase assays validating the direct targets *KRAS* and *SLC39A7* [49,60].	Proteinase K digestion of PG-EVLPs abolished macrophage M1 polarization and cytokine secretion, while DNase I/RNase I digestion did not, pinpointing vesicle proteins—rather than nucleic acids—as the functional cargo. Ultrasound-mediated structural disruption further abrogated these effects, indicating that intact vesicles delivering their protein cargo are required for macrophage activation [40].	PG-EVLPs lipids (Bligh–Dyer extract) served as an effective transfection reagent for ptc-*miR396f*, achieving *c-MYC* gene silencing, and DiD-labeled PG-EVLPs were preferentially taken up by C6 cells over EPC liposomes—supporting PG-EVLPs lipids as cross-kingdom delivery vehicle [25].	PG-EVLPs (0–10 µg/mL) dose-dependently mineralization via BMP-2/Smad1/5/9 signaling. (i) Free ginsenoside Rg3 at a concentration equivalent to its content in PG-EVLPs showed weaker effects on ALP activity and mineralized nodules than intact vesicles. (ii) Heat-inactivated PG-EVLPs (100 °C, 30 min) retained partial but reduced mineralization—indicating that both heat-stable (ginsenosides) and heat-labile (protein) components contribute to the osteogenic effect.	Not reported
Evidence level	Preclinical (in vitro/animal); cross-kingdom RNAi mechanisms; no clinical evidence [14,25,60].	Preclinical (in vitro/animal); marker validation only; no human data [14,35,36].	Preclinical lipidomic profiling + cell functional experiments; no human data [25,30,35].	Preclinical in-vitro/multiple animal disease models; PG-EVLPs superior vs free individual ginsenosides; no human data [15,50,56,57,66].	No study on the qualitative or quantitative analysis of polysaccharides in PG-EVLPs has been reported.

**Table 4:** Therapeutic diseases and mechanisms of action.

Disease Category	PG-EVLPs Source	Preparation	Mechanism of Action	Experimental Model	Key Findings	Evidence Level
Inflammatory diseases	Ginseng root [17,26,28,44,57,60]; Ginsengstem-leaf EVs [36].	Native: dUC/sucrose-gradient isolation [17,26,28,44,57,60]. Modified: neutrophil-membrane engineering + electroporation of *miR-182-5p* [19]; PEGylation + HA (CD44) + curcumin loading [36]; exosome-liposome hybrid (Ge-Lip@IBP) in oxygen-carrying foam (TW-NP@PFP-O2) for rectal delivery [29].	RNase digestion abolished PG-EVLPs’ anti-inflammatory activity, whereas proteinase K did not, while *pgi-miR6135j* mimic recapitulated joint effects [60]. Modification functions: neutrophil-membrane camouflage targeted inflamed lung [19]. HA-CD44 accumulation at arthritic joints + PEG-prolonged circulation [36]. (4)Rectal foam gave sustained antipyretic release + perfluorocarbon oxygen supply, mitigating pneumonia cytokine storm [29]. Biodistribution: intestine with macrophage co-localization [28]; joint tissue of CIA mice (DiL) [60]; rectal retention + systemic distribution (DiR) [29]. Only PG-EVLPs (ibuprofen 10 mg/kg rectal: AUC_0−24h_ 173.78 ± 27.06 µg·h/mL, Cmax 13.75 ± 1.41 µg/mL, T½β 10.03 ± 1.37 h, MRT 14.77 ± 1.97 h, superior to suppository/i.v.); others not reported [29].	LPS-induced sepsis [57]; sepsis-induced ALI [19]; DSS-induced colitis [17,28,44]; collagen-induced arthritis [36,60]. LPS-challenged pediatric pneumonia + SD-rat PK study [29].	Suppressed IL-1β/IL-6/TNF-α [19,26,28,57]; alleviated joint swelling and synovitis [44,60]; restored colonic barrier [17,28,44].NanoAid relieved pneumonia cytokine storm, reduced fever and corrected hypoxemia with superior [29].	Preclinical (cell + animal); no human trials.
Neoplastic diseases	Ginseng root [25,33,40,46]; stem/root EVs with miRNA cargo [46]. Modified: CDDP-loaded G-CDDP [37]; hydrogel-embedded GENs [47]; tumor-membrane hybrid vaccine HM-NPs [58]; CuPBA-mineralized iRGD [52]; ICG/CMSN@GsE [27]; HA-coated IGNITE [85]. Ginsenosides: free Rh2 [50].	Native: dUC/gradient isolation [25,33,40,46,81]. Modified: CDDP incubation + ultrafiltration [37]; microfluidic exosome-liposome fusion [47]; tumor-membrane hybrid vaccination [58]; in-situ CuPBA mineralization + iRGD [52]; CMSN core-shell + ICG [52]; HA coating [85]. Ginsenoside: free Rh2 without carrier [50].	Control design: G-CDDP vs free CDDP at equal CDDP (0.237 µg/mL), equivalent cytotoxicity at 12.66-fold lower dose [37]. Ginsenoside control: free Rh2 (50 µg/mL) ± cisplatin showed intrinsic anti-NSCLC activity [50]. Modification functions: iRGD tumor penetration + CuPBA cuproptosis/ferroptosis + VM inhibition [52]; TAMs (M2-like) targeting by GsE coating [86]; hybrid-membrane vaccine amplified DC/lymph-node antigen presentation [63]; HA coating improved storage stability [85]; native GENs crossed BBB to target glioma [25]; gEV miRNA profiles linked to anti-proliferative/anti-migratory effects [46]. Biodistribution: tumor-site targeting (DiD) [37]; liver accumulation in HCC [52]; glioma accumulation [25].	U-87MG glioma xenograft [37]; B16F10 melanoma [40,58]; A549/H1299 + LLC syngeneic NSCLC [33,50]; hepatocellular carcinoma [52]; orthotopic glioma [25] 4T1 breast cancer [47]; CT26 colon cancer [46]; cold-tumor model [81].	Suppressed tumor growth/metastasis [25,33,37,40]; chemotherapy sensitization by Rh2 [50]; VM inhibition [52]; TME remodeling + antitumor immunity [58,81,85]; PD-L1-mediated immune evasion attenuated [33].	Preclinical (cell + animal); no human trials.
Organ injury	Ginseng root [16,42,48,57]. Modified: VHPKQHR-targeted IL-6-siRNA exosomes [27]; neutrophil-membrane N-exo-miR182-5p [19]; ginseng-immune hybrid vesicles [45].	Native: dUC/TFF isolation [16,42,48,57]. Modified: VHP peptide conjugation + IL-6 siRNA loading [27]; neutrophil-membrane engineering [19]; ginseng-immune membrane fusion [45].	Cargo attribution: RNase/proteinase K digestion indicated RNA cargo as a major contributor in liver fibrosis [16]. Modification functions: VHP peptide targeted siRNA to injured hepatic endothelium [27]; neutrophil-membrane camouflage directed vesicles to inflamed lung in sepsis ALI [19]; immune-hybrid membrane conferred pulmonary endothelium targeting and restored mitochondrial homeostasis [45]. Biodistribution: liver (IRI/fibrosis) [16,27]; lung [19,45]; heart [42]; multi-organ in sepsis [57].	Alcohol-induced liver injury [48]; hepatic ischemia-reperfusion injury [27]; MASLD/ALD liver fibrosis [16]; sepsis and sepsis-induced ALI [19,57]; CDDP-induced cardiotoxicity [42]; lung ischemia-reperfusion injury [45].	Alleviated hepatocyte/cardiomyocyte/pulmonary injury; mitigated fibrosis; restored organ function [16,27,42,45,48]; attenuated sepsis-driven multi-organ damage [19,57].	Preclinical (cell + animal); no human trials.
Bone metabolic diseases	Ginseng-root [15,49,55]; red-ginseng RGNVs [66] Modified: PEG + HA Cur@EVs-PH (RA) [36]. Ginsenosides: free Rb1/Rg1 [15]; free Rg3 (equal amount) [66].	Native: sucrose-gradient purification [15]; dUC isolation [49,55]; multistep ultracentrifugation of red ginseng [66]. Modified: PEG + HA (CD44) + curcumin [36]. Ginsenosides: free Rb1/Rg1 [15]; Rg3 at a concentration equivalent to its content in the RGNV treatment [66].	Control design (native vs ginsenosides): GDNs (LC-MS: Rb1 64.5%/Rg1 31.1%) inhibited osteoclast differentiation more strongly than free Rb1/Rg1 alone or 7:3/5:5 mixtures [15]; RGNVs promoted osteogenesis/mineralization more strongly than equal-amount free Rg3 [66]; heat inactivation (100 °C, 30 min) only partially reduced RGNV activity → heat-stable ginsenosides + heat-labile proteins jointly contribute [66]. Cargo attribution: *miR-2118* mimic recapitulated and inhibitor reversed anti-ferroptotic phenotype; dual-luciferase verified SLC39A7 targeting [49].	LPS-induced bone-resorption model [15]; OVX osteoporosis mouse [55,66]; OA chondrocyte ferroptosis (ACLT) [49]; collagen-induced arthritis [36,60].	Suppressed bone resorption; improved bone micro-architecture [15,55,66] restored bone volume and BMD [55,66]; protected chondrocytes from ferroptosis [49]; alleviated RA joint destruction [36,60].	Preclinical (cell + animal); no human trials.
Skin related diseases	Ginseng root/callus/stem-leaf [20,30,35,53,56]. Modified: EGCG pH/ROS hydrogel [38]; CMCS/OHA hydrogel [34]; microneedle Pro@Gin-Exo [51]; hydrogel microneedle + PDA@PEGs [59]; microneedling protocol [87]; ROS-responsive DNA hydrogel + NO [54]; microneedling clinical protocol [88]. Ginsenosides: free Re/Rg1/Rb1/Rc (iso-content) [56].	Native: juice-squeezing/gradient isolation [20,22,30,35,53,56]. Modified: EGCG-loaded pH/ROS-responsive hydrogel [38]; CMCS/OHA hybrid hydrogel [34]; microfluidic membrane-fusion Pro@Gin-Exo + microneedle roller [51]; hydrogel microneedles [59]; MTS microneedling [87,88]; AIEgen-modified ROS-responsive DNA hydrogel + L-Arg/NO [54]. Ginsenosides: Re (3.8)/Rg1 (3.5)/Rb1 (1.1)/Rc (0.7 µg/mL), iso-content to GrDENs (2 × 10^9^ particles/mL) [56].	Control design (native vs ginsenosides): GrDENs provided the most comprehensive UV/H_2_O_2_ protection, while only Re alone significantly inhibited MMP2 among the four iso-content-free ginsenosides [56]. Modification functions: hydrogel/microneedle platforms gave local sustained/controlled release [34,38,51,54,59,87]; pH/ROS-responsive hydrogel released EGCG and GEX in response to oral-ulcer microenvironment [38]; AIEgen enabled fluorescence imaging + NO gas therapy in infected wounds [54]. Biodistribution: topical/local delivery via hydrogels and microneedles [34,38,51,54,59].	Diabetic-wound model [30,53]; full-thickness skin defect [20,54]; UVB-irradiated HaCaT/keratinocytes [56]; UVB skin senescence [35]; vitiligo mouse [59]; burn model [34]; photoaging [51]; diabetic oral-ulcer model [38]; PIH case reports [87,88].	Accelerated wound closure and angiogenesis [20,30,53,54]; anti-photoaging and MMP2 suppression [51,62]; anti-senescence [35]; repigmentation in vitiligo [38]; oral-ulcer repair improved PIH [87,88].	Preclinical (cell + animal); limited human case reports [62,88].
Neurological & ocular diseases	Modified: spermidine-modified S-GEVs@siRNA [39]; fibrin-gel-loaded G-Exos [43]. Native: none with direct CNS/ocular application. Ginsenosides: none.	Modified: spermidine surface modification of ginseng EVs + *Alox12B* siRNA loading for nasal-to-brain delivery [39]; ginseng exosomes loaded into fibrin gel for controlled ocular release [43].	Modification functions: spermidine enabled olfactory-receptor TAAR-mediated endocytosis; intranasal delivery via olfactory nerve pathway bypassed the BBB, releasing siRNA into hippocampal neurons and downregulating *Alox12B* [39]; fibrin gel provided sustained local release to protect retinal ganglion cells and modulate microglial polarization in glaucoma [43]. Biodistribution: hippocampus via nasal-to-brain route [39]; ocular (retinal) local retention [43];	Radiation-induced brain injury (RBI) mouse model [39] glaucoma model with retinal ganglion cell (RGC) assessment [43].	Alleviated RBI-induced cognitive dysfunction via neuroprotective siRNA delivery [39]; promoted RGC survival in glaucoma [43].	Preclinical (cell + animal); no human trials.
Periodontal disease	Ginseng-roo Modified: thermosensitive hydrogel-loaded GEXs (GEXs@Gel) [41].	Native: dUC + sucrose-gradient isolation of GEXs [41]. Modified: loading into thermosensitive Schiff-base hydrogel (OHA-PBA + NH_2_-F127).	Modification function: thermosensitive hydrogel conferred local retention and sustained release in the periodontal pocket, overcoming rapid clearance of free exosomes and maintaining therapeutic concentration at the lesion [41] Biodistribution: local gingival/periodontal retention (hydrogel depot).	Rat/mouse periodontitis model [41].	Reduced periodontal inflammation; eliminated pathogenic biofilm; facilitated alveolar-bone reconstruction [41].	Preclinical (cell + animal); no human data.
Metabolic disease	Ginseng-root [14]; diabetic-wound PG-EVLP [30,53].	Native: tangential-flow-filtration (TFF) isolation (50 nm hollow fibre) [14] dUC/gradient isolation [30,53].	Preparation-based rationale: TFF isolation yielded high-purity PGEs (159.5 nm; 3.9 × 10^12^ particles/mL; removal of 63.6% soluble protein; 771-fold RNA enrichment; TET-8-positive), preserving intact bioactive cargo without harsh processing [14].	3T3-L1 preadipocytes; high-fat-diet metabolic model [14]; diabetic-wound models [30,53]	72.1% reduction of lipid accumulation (Oil Red O); inhibited adipogenic differentiation; improved gut microecology [14]; accelerated diabetic-wound healing [30,53].	Preclinical (cell + animal); no human trials.

## Data Availability

The datasets used or analyzed in the current study are available from the corresponding author upon reasonable request.

## Abbreviations

The following abbreviations are used in this manuscript:

Akt	Protein kinase B
ALT	Alanine transaminase
AMPK	Adenosine 5′-monophosphate-activated protein kinase
AST	Aspartate transaminase
Bax	Bcl-2-associated X protein
Bcl-2	B-cell lymphoma-2
BMP2/4	Bone morphogenetic protein 2/4
CCL5	C-C motif chemokine ligand 5
Cryo-EM	Cryogenic transmission electron microscopy
CXCL9	C-X-C motif chemokine ligand 9
DLS	Dynamic light scattering
DSS	Dextran sulfate sodium
EGCG	Epigallocatechin gallate
EGFR	Epidermal growth factor receptor
PG-EVLP	*Panax ginseng*-derived EV-like particles
GPX4	Glutathione peroxidase 4
GSH-Px	Glutathione peroxidase
HIF-1α	Hypoxia-inducible factor-1α
HSP70	Heat shock protein 70
HSP90	Heat shock protein 90
HUVEC	Human umbilical vein endothelial cell
IKK	IκB kinase
IL	Interleukin
KEAP1	Kelch-like ECH-associated protein 1
KRAS	Kirsten rat sarcoma viral oncogene homolog
LC-MS/MS	Liquid chromatography-tandem mass spectrometry
LPS	Lipopolysaccharide
miRNA	MicroRNA
M1	Classical activated macrophage
M2	Alternative activated macrophage
MDSC	Myeloid-derived suppressor cell
mRNA	Messenger RNA
NLRP3	NOD-like receptor family pyrin domain-containing 3
NOX4	NADPH oxidase 4
Nrf2	Nuclear factor erythroid 2-related factor 2
NTA	Nanoparticle tracking analysis
OVX	Ovariectomized
PC	Phosphatidylcholine
PE	Phosphatidylethanolamine
PI3K	Phosphatidylinositol 3-kinase
PEG	Polyethylene glycol
RANKL	Receptor activator of nuclear factor κB ligand
SEC	Size exclusion chromatography
SMAD	Small mothers against decapentaplegic
SM	Sphingomyelin
SOD	Superoxide dismutase
TAMs	Tumor-associated macrophages
TCF7	Transcription factor 7
TEM	Transmission electron microscopy
TGF-β	Transforming growth factor-β
TIMP2	Tissue inhibitor of metalloproteinase 2
TLR4	Toll-like receptor 4
TNF-α	Tumor necrosis factor-α
Treg	Regulatory T cell
Th17	T helper 17 cell
UF	Ultrafiltration
USP22	Ubiquitin-specific protease 22
